# Genomic Comparison of *Lactobacillus helveticus* Strains Highlights Probiotic Potential

**DOI:** 10.3389/fmicb.2019.01380

**Published:** 2019-06-26

**Authors:** Alessandra Fontana, Irene Falasconi, Paola Molinari, Laura Treu, Arianna Basile, Alessandro Vezzi, Stefano Campanaro, Lorenzo Morelli

**Affiliations:** ^1^Department for Sustainable Food Process – DiSTAS, Università Cattolica del Sacro Cuore, Piacenza, Italy; ^2^Department of Biology, University of Padua, Padua, Italy; ^3^CRIBI Biotechnology Center, University of Padua, Padua, Italy

**Keywords:** lactic acid bacteria, *Lactobacillus helveticus*, bile salts tolerance, exopolysaccharides, antibiotic resistance, comparative genomics, probiotics

## Abstract

*Lactobacillus helveticus* belongs to the large group of lactic acid bacteria (LAB), which are the major players in the fermentation of a wide range of foods. LAB are also present in the human gut, which has often been exploited as a reservoir of potential novel probiotic strains, but several parameters need to be assessed before establishing their safety and potential use for human consumption. In the present study, six *L. helveticus* strains isolated from natural whey cultures were analyzed for their phenotype and genotype in exopolysaccharide (EPS) production, low pH and bile salt tolerance, bile salt hydrolase (BSH) activity, and antibiotic resistance profile. In addition, a comparative genomic investigation was performed between the six newly sequenced strains and the 51 publicly available genomes of *L. helveticus* to define the pangenome structure. The results indicate that the newly sequenced strain UC1267 and the deposited strain DSM 20075 can be considered good candidates for gut-adapted strains due to their ability to survive in the presence of 0.2% glycocholic acid (GCA) and 1% taurocholic and taurodeoxycholic acid (TDCA). Moreover, these strains had the highest bile salt deconjugation activity among the tested *L. helveticus* strains. Considering the safety profile, none of these strains presented antibiotic resistance phenotypically and/or at the genome level. The pangenome analysis revealed genes specific to the new isolates, such as enzymes related to folate biosynthesis in strains UC1266 and UC1267 and an integrated phage in strain UC1035. Finally, the presence of maltose-degrading enzymes and multiple copies of 6-phospho-β-glucosidase genes in our strains indicates the capability to metabolize sugars other than lactose, which is related solely to dairy niches.

## Introduction

Lactic acid bacteria (LAB) play a key role in the production of various fermented foods, as well as in diverse environments, such as soil and the human gut, which is often considered a reservoir of potential novel probiotic strains. *Lactobacillus helveticus* was first described by Orla-Jensen in 1919 as *Thermobacterium helveticum*, in which the prefix “thermos” referred to the high temperature used for the production of Emmental, the initial isolation source of the bacterium (Naser et al., 2006). In addition to Swiss-type cheeses, strains belonging to this species are present in natural whey cultures from Italian long-ripened cheeses (e.g., Parmigiano Reggiano and Grana Padano) (Giraffa et al., 2000), strongly suggesting that the primary habitat of this species is the dairy environment. However, some strains of *L. helveticus* contain specific probiotic features in their genome. For example, an immune protection effect related to S-layer proteins has been demonstrated in *L. helveticus* M92 and NS8 (Beganović et al., 2011; Rong et al., 2015). Additionally, high production of exopolysaccharides (EPSs) and bacteriocins in the MB2-1 and KLDS1.8701 strains, respectively, have been studied (Li et al., 2015a, b). In addition, the inhibition of *Campylobacter jejuni* invasion was demonstrated for *L. helveticus* R0052 (Wine et al., 2009), whereas the MTCC 5463 strain possesses genes for adhesion and aggregation, including mucus-binding proteins (Senan et al., 2015b). Furthermore, a close relatedness exists between *L. helveticus* DPC 4571 and *Lactobacillus acidophilus* NCFM found in the gastrointestinal tract (GIT), which exhibit 98.4% sequence identity for the 16S rRNA gene (Callanan et al., 2008). An important environmental factor for bacterial niche specialization is the type of sugar available (Slattery et al., 2010). For instance, lactose is the main sugar present in the dairy niches, whereas maltose is typically located in environments where starch metabolic by-products are present, such as the gut. The presence of the enzyme maltose-6-phosphate glycosidase, as well as multiple copies of glucosidase genes, can be putative indicators of a gut-adapted microorganism (Slattery et al., 2010). Most common probiotic strains contain two copies of the enzyme α-1,6-glucosidase (Cremonesi et al., 2012; Møller et al., 2012, 2014). However, only one copy of this enzyme has been found in the dairy strain *L. helveticus* DPC 4571 (Slattery et al., 2010).

Another important element allowing adaptation to a certain niche is the capacity to deal with stressful conditions, such as those present in the gut environment. For example, the survival at the low pH of the stomach is fundamental, as well as the tolerance to bile salts and the presence of a functional bile salt hydrolase (BSH). Bile acids originate from cholesterol and can be found in a conjugated form with either glycine or taurine. The toxicity toward bacterial cells relies on their surfactant-like nature, which induces intracellular acidification or disrupts cell membranes (Begley et al., 2005). Deconjugation of bile salts by BSH activity increases their recovery *via* passive absorption through the colonic epithelium (Ridlon et al., 2006). A frame-shifted, non-functioning *bsh* has been found in *L. helveticus* DPC 4571 (Slattery et al., 2010). In contrast, the presence of a bile acid-inducible operon, containing one *bsh* gene and two choloylglycine hydrolases, has been highlighted in *L. helveticus* MTCC 5463 (Senan et al., 2015b). Nonetheless, other genes are involved in the mechanisms underlying bile salt tolerance. For example, in the probiotic strain *L. acidophilus* NCFM, among the genes induced in the presence of bile are some major facilitator superfamily (MFS) members, permease, and ATPase subunits of the ABC transporters (Pfeiler and Klaenhammer, 2009). All of these genes were annotated as members of the multidrug resistance (MDR) family, which plays a role in defending against inhibitory compounds by ejecting a wide variety of substrates from the cell, such as antibiotics, bile salts, and peptides.

In addition to bile salt tolerance and hydrolyzation, the ability to colonize the GIT by forming biofilms is also fundamental (Branda et al., 2005). In this context, EPSs play a key role, contributing to the structural diversity of the cell wall of *Lactobacillus* spp. (De Vuyst and Degeest, 1999). Moreover, the presence of other cell surface factors, such as S-layer proteins, which are not present in all *Lactobacillus* spp., can promote adherence and immunostimulation mechanisms and be involved in competitive pathogen exclusion (Åvall-Jääskeläinen and Palva, 2005; Lebeer et al., 2008). For instance, surface-layer extracts from *L. helveticus* R0052 have been shown to inhibit the adhesion of *Escherichia coli* O157:H7 to epithelial cells (Johnson-henry et al., 2007). S-layer-related genes have also been found in other strains of *L. helveticus*, namely, CNRZ 892, MIMLh5, M92, NS8, and MTCC 5463 (Callegari et al., 1998; Beganović et al., 2011; Taverniti et al., 2013; Rong et al., 2015; Senan et al., 2015b).

Considering the safety profile characterizing a putative probiotic strain, analysis of the antibiotic resistance pattern is important. A wide range of antibiotic resistance has been found in many *L. helveticus* strains isolated from dairy products, including Grana Padano and Provolone cheese starters (Fortina et al., 1998; Frece et al., 2009). Specifically, resistance to rifampicin, chloramphenicol, kanamycin, lincomycin, streptomycin, polymixin B, and rifamycin has been highlighted.

According to the literature, many of the 51 genomes of *L. helveticus* deposited possess proven probiotic capability, namely, R0052 (Mohammadi et al., 2018), KLDS1.8701 (Li et al., 2017), CAUH18 (Yang et al., 2016), MB2-1 (Li et al., 2015a), MTCC5 463 (Senan et al., 2015a), H9 (Chen et al., 2015), M92 (Beganović et al., 2013), and D75 and D76 (Roshchina et al., 2018). Specifically, R0052 is characterized by the production of mucus-binding proteins and surface-layer proteins; CAUH18, by EPS formation and cell aggregation properties; and M92, H9, D75, and D76, by their proteolytic activity and bacteriocin production. However, as the remaining strains may have unidentified phenotypic probiotic features, all of the 51 available *L. helveticus* strains were included in the comparative analysis described in the present study. The observed adaptation of *L. helveticus* strains to different ecological niches, such as the gut and dairy, suggests the need for more in-depth investigation at both the genomic and phenotypic levels, which can be useful for gaining insights into the evolutionary history of this species. Moreover, the demand for new interesting strains for industry-driven applications in cheese ripening and health-promoting products opens further perspectives for *L. helveticus* (Giraffa, 2014).

In the present study, the phenotypes and genotypes of six *L. helveticus* strains isolated from natural whey cultures were analyzed to highlight specific features for possible application as probiotics. Specifically, EPS production, S-layer-related genes, low pH and bile salt tolerance, BSH activity, and antibiotic resistance were evaluated. In addition to the search for properties of gut-adapted strains, a comparative genomic investigation was performed between the newly sequenced strains and the 51 publicly available genomes of *L. helveticus*.

## Materials and Methods

### Whole Genome Sequencing of *L. helveticus* Strains

Genomic DNA of six *L. helveticus* strains (UC1035, UC1266, UC1267, UC1275, UC1285, and UC3147) was extracted with the E.Z.N.A.^®^ Bacterial DNA Kit (Omega Bio-tek, United States). The quality of the extracted DNA was checked by agarose gel electrophoresis (0.8%) and then quantified with the Qubit fluorometer (Life Technologies, Carlsbad, CA, United States). Genomic DNA was sequenced using the Illumina MiSeq technology (2 × 150 bp). Reads were filtered and assembled with CLC Genomics workbench v. 5.1 (CLC Bio, Aarhus, DK, United States) using CLC’s *de novo* assembly algorithm, using a k-mer of 63 and a bubble size of 60, as previously described (Zhu et al., 2019). Only scaffolds longer than 1 kb were considered for further analyses.

### Comparative Genome Analysis

Fifty-one *L. helveticus* strain genomes were downloaded from NCBI microbial genome database^1^ (December 2018). Genome metrics (e.g., genome size, N50, number of scaffolds, etc.) were determined using CheckM (v1.0.7) (Parks et al., 2015). Gene prediction and annotation were performed using Prokka (v1.12) (Seemann, 2014) trained on *Lactobacillus* annotations deposited in NCBI database. Annotation was refined with EggNOG (v4.5.1) using eggNOG-mapper (Huerta-Cepas et al., 2016) using as input the protein sequences predicted with PROKKA. All the *L. helveticus* genomes were uploaded to the RAST server and annotated using SEED and the RAST gene caller (Overbeek et al., 2014). Annotations obtained from PROKKA and RAST in tabular format and genbank format were uploaded and made available in sourceforge^2^.

Annotation results were downloaded, and using in-house developed perl scripts (Treu et al., 2018), the number of genes present in each SEED category was determined considering both “first” and “second” level (Supplementary Data S2). Pangenome was predicted with Roary (v3.11.2) (Page et al., 2015) using as input the annotation files previously generated, and results were visualized using the script “roary_plots.py.” “Unique” and “new” genes derived from pangenome analysis were determined using create_pan_genome_plots.R software of the Roary package. The core genomes of *L. helveticus* were aligned using Parsnp (v1.2) (Edgar, 2004; Bruen et al., 2006; Price et al., 2010; Treangen et al., 2014) producing variant (SNP) calls and core genome phylogeny. An additional verification of the genes present in the six strains sequenced in the current project was performed independently from the assembly and considering all the pangenome sequences obtained from Roary. More specifically, a representative gene sequence was collected for each gene cluster giving priority to the sequences of the complete strains downloaded from the NCBI database; shotgun Illumina reads obtained for each strain were aligned on the “pangenome database” using Bowtie 2 (v2.2.4) (Langmead and Salzberg, 2012), and the coverage of each gene was determined using pileup.sh software of the BBTools package (Supplementary Data S2). The files in “newick tree” format obtained with Prokka and Parsnp were re-rooted on strain KLDS1.8701 and decorated using iTol (Letunic and Bork, 2016). Genome sequences were aligned using progressive MAUVE software to identify strain-specific regions and their presence in the genome (Darling et al., 2010). The strain CAUH18 deposited in RefSeq was used as reference for genome comparison for Supplementary Figure S1. Presence of antibiotic resistance genes (ARG), prophages, bacteriocins, and plasmids was evaluated in the strains sequenced in the present study. ARG analysis was performed using RGI (v3.2.1) (Jia et al., 2017) with parameter “–loose_criteria = no.” Integrated prophages were investigated using PHASTER (Arndt et al., 2016). The presence of Clustered Regularly Interspaced Short Palindromic Repeats (CRISPRs) was evaluated with CRISPRCasFinder (Couvin et al., 2018) (Supplementary Data S3). Bacteriocins were tested using Bagel4 (de Jong et al., 2006). Presence of plasmid sequences was evaluated using plasmidSPAdes (v3.13.0) (Antipov et al., 2016). Presence of BSH and penicillin-V acylase (PVA) genes was verified using hmmsearch (Eddy, 2011); briefly, protein sequences of BSH and VPA were recovered from NCBI database, aligned using clustalw (v2.1) (Thompson et al., 2002), a hidden Markov model was built with hmmbuild (v3.1b1) (Eddy, 1998), and protein sequences obtained from Prokka were analyzed using hmmsearch (Supplementary Data S3).

### EPS Production Evaluation

The ability to produce EPS was tested by inoculating our six isolates in de Man Rogosa Sharpe (MRS)-lac agar in which glucose was replaced by 2% of lactose (Torino et al., 2005). Ropy phenotype was examined by picking the colonies with a sterile loop and observing the formation of a filament when the loop was lifted (Ruas-Madiedo and de los Reyes-Gavilán, 2005).

### Bile Salts and Low pH Tolerance Assays

Bile salt tolerance was assessed by cultivating the bacterial strains in the presence of increasing concentrations of the following bile salts: glycocholic acid (GCA), glycodeoxycholic acid (GDCA), taurocholic acid (TCA), and taurodeoxycholic acid (TDCA). The concentrations tested were 0.01, 0.1, 0.2, 0.5, 1.0, and 2.0% w/v for each compound. Briefly, overnight cultures were centrifuged, washed twice with PBS, and adjusted to a final Abs_600_ of 1. Ten microliters of the cell suspension was inoculated in a 96-well plate containing 190 μl of MRS added with different concentrations of each bile salt and then incubated for 24 h at 45°C. After incubation, Abs_600_ was measured and the results were expressed as the percentage of growth in the presence of bile salts compared to the control grown without the addition of any compound (Shinoda et al., 2001).

The test for tolerance to low pH was carried out following the protocol of Prasad et al. (1999) with minor changes. Strains were cultivated overnight at 45°C, and then 0.2 ml was centrifuged, washed twice with 1 ml of NaCl 5 g/L, and resuspended in 2 ml of an acid solution composed of NaCl 5 g/L and 25 mM glucose at pH 3 to have approximately 10^8^ CFU/ml. A 96-well plate was filled with 200 μl of each acid solution inoculated with the different strains and incubated at 45°C. Aliquots of 100 μl of each sample were collected at time 0, 60, 120, 180, and 300 min after incubation and used to make the bacterial counts.

### BSH Activity

Bacterial strains were grown overnight at 45°C in 50 ml of MRS, centrifuged for 10 min at 10,000 × *g* at 4°C, washed twice with 0.1 M sodium-phosphate buffer pH 6.8, and resuspended in the same buffer. The obtained suspension was sonicated for 60 s using a CV17 sonicator (VibraCell, Sonics and Materials Inc., Newtown, CT, United States) and centrifuged to remove cell debris (Noriega et al., 2006) in order to obtain cell-free extracts. Quantitative determination of the BSH activity was calculated according to the two-step method described by Tanaka et al. (1999). In the first reaction, conjugated primary/secondary bile salts were incubated in a reaction mix with the different cell-free extracts, to achieve the release of amino acids from the bile salts. These amino acids were quantified in the second reaction as follows: 5 μl from the BSH reactions diluted five times with 0.5 M sodium-citrate buffer pH 5.5 was mixed with 110 μl of the ninhydrin reagent, incubated 14 min at 97°C in a PCR Thermal Cycler, and cooled down to 4°C. Standard curves were made with 5 μl of glycine in place of the sample. Abs_570_ was measured after 30 min in an Epoch^TM^ Spectrophotometer (Biotek, Winooski, VT, United States). BSH activity was expressed in U/ml, since one unit of BSH activity was defined as the amount of enzyme that liberated 1 μmol glycine from GDCA per minute (Jiang et al., 2010).

### Antibiotic Resistance Assay

The susceptibility profiles of the isolated strains to gentamycin, kanamycin, streptomycin, neomycin, tetracycline, erythromycin, clindamycin, and chloramphenicol were determined by broth microdilution using VetMIC plates for LAB (VetMIC Lact-1; National Veterinary Institute, Uppsala, Sweden). The inoculum for the test was prepared by picking colonies from fresh cultures grown on MRS agar plates (Difco, Detroit, MI, United States) and suspending them in sterile saline solution (NaCl 9 g/L) to reach an optical density corresponding to McFarland standard 1. The suspension was then diluted 1:1,000 in LAB susceptibility test medium (LSM), composed of 90% of Iso-Sensitest broth and 10% of MRS broth; 100 μl of the final bacterial suspension was added to each well of the VetMIC plates. The plates were then incubated at 37°C for 48 h in anaerobic conditions. The minimum inhibitory concentration (MIC) was defined as the lowest antibiotic concentration at which no growth was observed (Huys et al., 2010). Results were compared to the cutoff values edited by EFSA (European Food Safety Authority, 2012). *L. helveticus* DSM 20075 was used as reference strain.

### Statistical Analysis

To determine significant differences (*P* < 0.05) between the strains in relation to bile salt concentration, two-way analysis of variance (ANOVA) followed by Bonferroni multiple comparisons test was performed. One-way ANOVA followed by Tukey’s multiple comparisons test was carried out to compare BSH results. Both analyses were implemented using GraphPad Prism5 (GraphPad Software, La Jolla, CA, United States).

## Results and Discussion

The average genome size and GC content of the six newly sequenced *L. helveticus* strains were 1.9 Mb and 36.7%, respectively (Supplementary Data S1). Gene finding and annotation resulted in an average of 2,090 coding sequences (CDSs), with a coding density of 84.7%.

### Comparative Genomics Analyses

**FIGURE 1:**
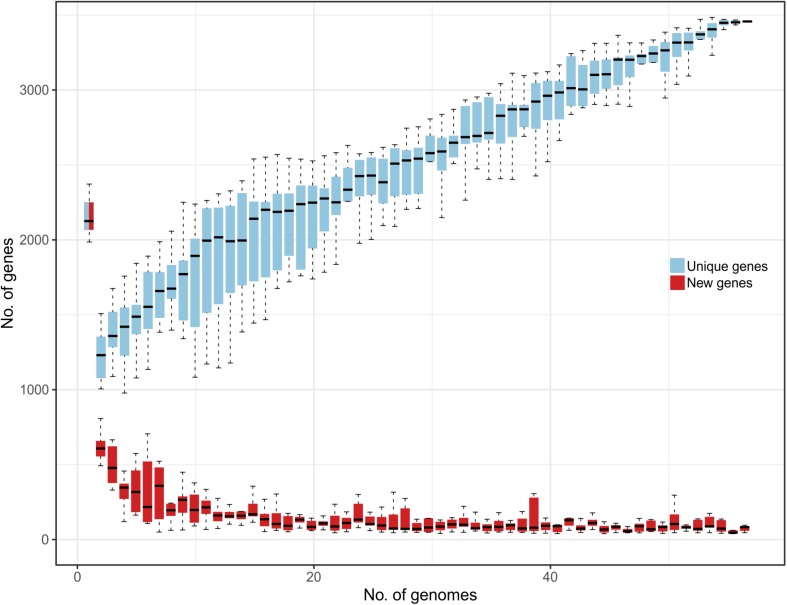
Representation of *L. helveticus* gene content reporting how the pan genome varies as genomes are added in random order to the analysis. In the *y* axes, the number of genes is reported, while in the *x* axes, the number of strains considered is shown. Both unique genes (azure) and new genes (red) are reported.

Comparative genomic analysis evidenced that the *L. helveticus* pangenome can be considered as “open” since nearly 30 new genes are continuously added for each additional genome considered (Figure 1). This suggests a remarkable range of phenotypic variability between strains conferred by the presence of a very flexible genetic content and the presence of strain-specific genes (unique) on each genome. This genomic heterogeneity and high number of publicly available *L. helveticus* strains hampered a graphical representation of all the aligned genomes. Therefore, an additional investigation of the pangenome was performed to identify the entire set of strain-specific genes. Computational mining of genome sequences aimed to favor the selection of strains for biotechnological use (probiotic potential), to investigate niche association, and to study phylogenetic correlation. The 57 genomes were grouped in terms of strain isolation and geographic localization to test for specific genome associations and functional gene groups. The defined niche categories based on geographical localization were as follows: Canada (*n* = 1), China (*n* = 7), Croatia (*n* = 1), Europe (*n* = 2), France (*n* = 6), India (*n* = 1), Italy (*n* = 11), Russia (*n* = 2), Swiss Confederation (*n* = 20), Tajikistan (*n* = 1), and United States (*n* = 5). According to the isolation source, strains were classified as follows: commercial dietary supplement (*n* = 5); dairy product (*n* = 27), divided into cheese, fermented milk, and raw milk; human (*n* = 3); industrial dairy starter (*n* = 2); malt fermentation (*n* = 1); natural whey culture (*n* = 14); and “not available” (*n* = 5) (Supplementary Data S1).

**FIGURE 2:**
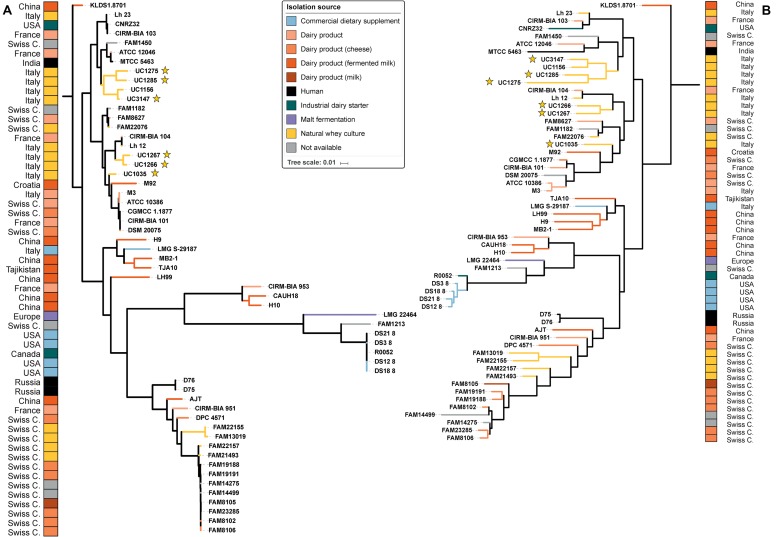
Phylogenetic trees of the 57 *L. helveticus* strains. The six strains newly sequenced in the present work are highlighted with a yellow star. The color choice depicts the isolation source of each strain, and the corresponding country of isolation is reported on the two external columns. Tree based on SNPs identified by Parsnp among the strains **(A)**. Tree based on orthologous genes found by Roary among the strains **(B)**. The length of each branch is proportional to the number of SNPs and orthologs found, respectively.

From the phylogenetic tree, our strains were divided into two clusters based on SNPs (Figure 2A): the first was composed of UC1275, UC1285, and UC3147, and the second was composed of UC1035, UC1266, and UC1267. The former cluster also comprises FAM1450, ATCC 12046, MTCC 5463, and UC1156. All of the strains in this group were isolated in Europe, except MTCC 5463, which was isolated in Asia. MTCC 5463 is also an outlier in regard to the isolation source, as all strains in the cluster are of dairy origin but MTCC 5463 was isolated from the vaginal mucosa. The second cluster consists of CIRM-BIA 104, Lh12, M92, M3, ATCC10386, CGMCC 1.1877, CIRM-BIA 101, and DSM 20075 in addition to the already mentioned strains. This cluster is more homogeneous than the other one because all of the strains were isolated in Europe and derived from a dairy environment. In both clusters, a probiotic strain is present, namely, MTCC 5463 in the first cluster (Senan et al., 2015b) and M92 in the second cluster (Beganović et al., 2011). Notably, all of the strains maintained the same distribution among the clusters considering both whole genome SNPs and the orthologous gene content (Figure 2B).

**FIGURE 3:**
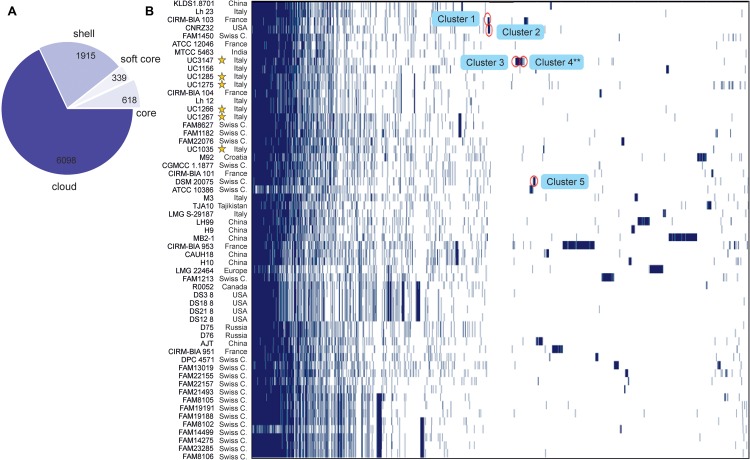
Gene content comparison of the 57 considered *L. helveticus* strains. **(A)** The number of genes belonging to the core, the soft core, the shell, or the cloud of the *L. helveticus* species is pictured as a pie chart. The six strains newly sequenced in the present work are highlighted with a yellow star. **(B)** The matrix shows genes typical of each strain and those conserved in all.

The Roary pangenome pipeline succeeded in identifying more than 8,000 different orthologous groups of proteins that, in relation to their distribution in the 57 strains, were organized into four different classes according to the number of strains sharing each orthologous group of proteins: “Core” (57 or 56 strains), “Soft-core” (54 or 55 strains), “Shell” (8–53 strains), and “Cloud” (less than eight strains) (Figure 3A). In the Roary analysis, some gene clusters were associated with the newly sequenced strains (Figure 3B). These gene clusters were inspected to understand which peculiarity they bestow on each strain; the most interesting are highlighted in Figure 3B. Specifically, five gene clusters were identified. Cluster 1, which was identified in UC1266 and UC1267, is characterized by the presence of enzymes belonging to the Shikimate pathway responsible for folate and aromatic amino acid (phenylalanine, tyrosine, and tryptophan) biosynthesis (Herrmann and Weaver, 1999). The production of B vitamins, such as folate (vitamin B_9_), by some strains of lactobacilli is considered to have a beneficial effect on the host in the case of vitamin deficiency (Wegkamp et al., 2004; Santos et al., 2008). Cluster 2, which was identified only in UC1267, is characterized by the presence of GARS, a mono-functional enzyme involved in purine biosynthesis (Kanai and Toh, 1999). Cluster 3, which was identified in UC1035, is typified by poly-gamma-glutamate (PGA) biosynthesis proteins. PGA allows bacteria to survive at high salt concentrations and may also be involved in virulence (Candela and Fouet, 2006). Cluster 4, which was also found in UC1035, is composed of phage proteins, the ArpU family of phage transcriptional regulators and holins (Wang et al., 2000). The analysis revealed the presence of a putative integrated prophage that was not identified by PHASTER. In the alignment obtained using Mauve software, we indeed identified a 40,500-bp strain-specific region in UC1035, absent in the reference genome (CAUH18), as shown in Supplementary Figure S1 (region 3). Finally, Cluster 5, which was specific to UC1285, is characterized by enzymes involved in aromatic compound catabolism.

### Functional Categories Related to Probiotic Capabilities

**FIGURE 4:**
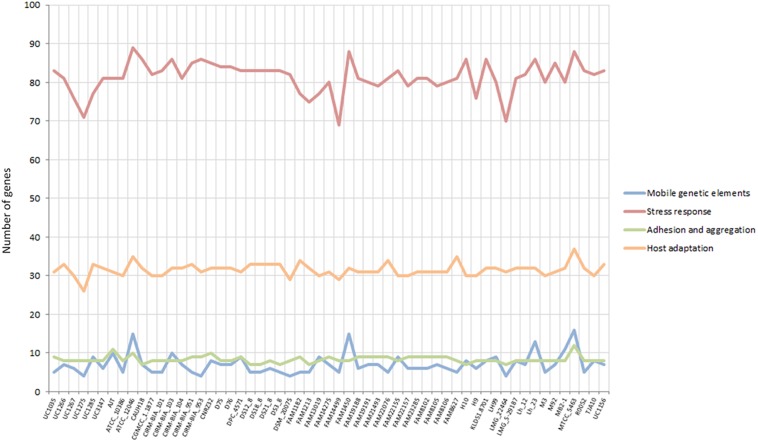
Gene content in the considered categories for the 57 investigated *L. helveticus* strains according to Roary.

Some specific features considered crucial for a gut-adapted microorganism were investigated more deeply at the genome level in order to assess the potential probiotic capabilities of each strain. Specifically, the presence of mobile genetic elements, epithelial adherence and aggregation features, stress response mechanisms, and host adaptation-related genes were evaluated. Considering the “mobile genetic elements” and “adhesion and aggregation” categories, the *L. helveticus* strains were comparable in terms of gene content, with seven and eight genes on average (Figure 4). The newly sequenced strains exhibited a similar number of adhesion and aggregation encoding genes compared to the other investigated strains (Figure 4). In relation to the “stress response” category, all 57 genomes have shown a high number of genes (ranging from 69 to 89; Figure 4). Of particular interest is the microbial capability to tolerate acidic pH and surfactant-like molecules, such as bile salts. Among our strains, UC1035, UC1266, and UC3147 had the highest number of genes in stress-related category (83, 81, and 81 genes, respectively; Figure 4). The second most abundant category in terms of gene content (from 26 to 37) was “host adaptation” (Figure 4). Considering all the strains, MTCC 5463 and ATCC 12046 genomes showed a high number of genes (37 and 35, respectively); among the newly sequenced, this feature characterizes UC1266 and UC1285 (33 genes each strain).

#### Mobile Genetic Elements

In bacteria, mobile genetic elements such as prophages, integrases, and insertion sequences (ISs) are primary contributing factors to genetic diversity and niche adaptation. Among the six strains sequenced in this study, UC1285 has the highest number of mobile genetic elements, containing nine genes (Figure 4). The six UC strains included genes encoding prophages and integrases (Supplementary Data S4), as previously identified in *L. helveticus* MTCC 5463 (Senan et al., 2015b). Specifically, the “prophage DNA packaging protein NU1,” a “phage-associated protein,” a “group II intron-encoded maturase,” a “putative integrase-recombinase,” and some “integrases” were determined (Supplementary Data S4). RAST annotation identified additional genes related to the “phages and prophages” category in all newly sequenced strains, with UC1035 and UC1266 having the highest number (14 and 11 genes, respectively). A similar profile was identified in CIRM-BIA 103, CIRM-BIA 104, CIRM-BIA 953, CNRZ32, some FAM strains (13019, 14275, 14499, 19188, 19191, 23285, 8102, and 8106), Lh12, Lh23, and MB2-1. Phage-related sequences were detected recently in other dairy isolates (i.e., FAM 8105, FAM 8627, and FAM 22155) (Schmid et al., 2018). The strains MTCC 5463, ATCC 12046, FAM 1450, and Lh 23 have the highest number of genes related to this category (ranging from 13 to 16 genes, some of them in multiple copies), including ISs (Supplementary Data S4). The presence of mobile genetic elements, such as ISs, is associated with the genomic instability of a strain because it promotes chromosomal rearrangements, such as deletions, duplications, and inversions (Mahillon and Chandler, 1998; Callanan et al., 2008). The lack of these features in the six *L. helveticus* strains analyzed in this study highlights a potentially high genomic stability, which is considered relevant for quality assurance of a probiotic strain (Sybesma et al., 2013).

#### Epithelial Adherence and Aggregation Features

Good adherence capacity is generally assumed to be a desirable trait for probiotic lactobacilli, as it can increase the gut residence time, improve efficiency of pathogen exclusion, and facilitate interactions with host cells. This latter feature is relevant for the protection of epithelial cells or immune modulation (Lebeer et al., 2008). Among the different factors involved in epithelial adherence and aggregation, EPSs generally play a role in the non-specific interactions of lactobacilli with abiotic and biotic surfaces. In this regard, EPSs seem to play a more specific role in the formation of microcolonies and biofilms (Branda et al., 2005).

Genome mining confirmed the experimental EPS assay performed only on the newly sequenced isolates but revealed also that six out of the 51 strains deposited (ATCC 10386, CGMCC 1.1877, CIRM-BIA 101, DSM 20075, M3, LMG 22464) seem not to have the genes coding for proteins involved in EPS biosynthesis. Five of these strains are phylogenetically related according to the SNP-based phylogenetic tree (Figure 2A). Unlike most of the examined strains, four of the newly sequenced strains are characterized by the presence of genes encoding for d-TDP-4-dehydro rhamnose reductase (i.e., UC1266, UC1275, UC1285, and UC3147), which converts dTDP-6-deoxy-L-mannose into dTDP-4-dehydro-6-deoxy-L-mannose. This suggests that EPSs in the newly sequenced strains could be synthesized from the precursor dTDP-rhamnose or the precursors converted through the Leloir pathway (UDP-glucose, UDP-galactose) (Barreto et al., 2005). However, previous studies found that some strains of *L. helveticus* produce EPSs using lactose as a substrate (Robijn et al., 1995; Stingele et al., 1997; Torino et al., 2001; Li et al., 2014). Among our isolates, the ropy phenotype was detected exclusively in UC1275 (data not shown). This phenotypic characteristic could be associated with the presence of two extra copies of d-TDP-4-dehydro rhamnose reductase, as well as the gene coding for dTDP-4-dehydrorhamnose 3,5-epimerase, which was also found in probiotic strain R0052. Moreover, four glycosyltransferase genes were specifically found only in the UC1275 strain, together with the epsIM gene (Supplementary Data S4).

**FIGURE 5:**
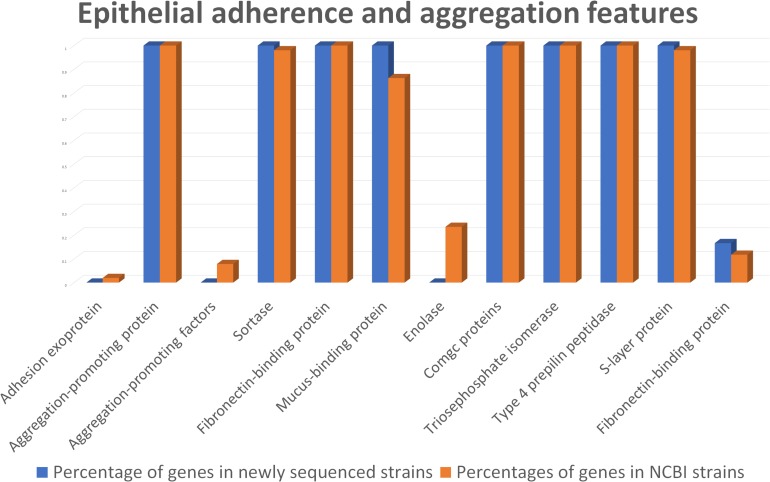
Comparison between the newly sequenced strains and publicly available strains genome mining for epithelial adherence and aggregation features.

According to genome mining, mucus-binding proteins were also identified (Figure 5 and Supplementary Data S4). These kinds of proteins have already been recognized for their importance in adhesion to the intestinal mucosa layer and may assist *L. helveticus* in binding to intestinal mucus, especially in the small intestinal tract, and in protecting epithelial cells. Together with EPSs, the production of mucus-binding proteins could indicate a putative use of this species as a probiotic, especially in the treatment of small intestinal bacterial overgrowth (SIBO), as suggested by Klopper et al. (2018). This class of proteins was found in all of the genomes of our strains, as well as in 44 out of 51 genomes under investigation, including the probiotic strains MTCC 5463, M92, and R0052 (Supplementary Data S4).

In the anchoring of mucus-binding proteins to the bacterial cell wall, sortases play a key role (Kleerebezem et al., 2010). Genes encoding sortase proteins were identified in all of the analyzed *L. helveticus* genomes, except FAM14499 (Supplementary Data S4).

Considering the S-layer proteins, their relevance to *Lactobacillus* spp. in supporting microbial persistence in the gut was assayed previously (Grosu-Tudor et al., 2016). These proteins can also interact with the cellular receptor dendritic cell-specific intercellular adhesion molecule-3-grabbing non-integrin (DC-SIGN; CD209) (Prado Acosta et al., 2016), preventing infection by pathogenic bacteria through a process of competitive exclusion (Zhang et al., 2017). Interestingly, genomic analyses revealed a higher number of S-layer genes (*n* = 10) in the UC1285 isolate, compared to the other *L. helveticus* strains, which contained six genes on average (Supplementary Data S4).

#### Stress Response Mechanisms

**FIGURE 6:**
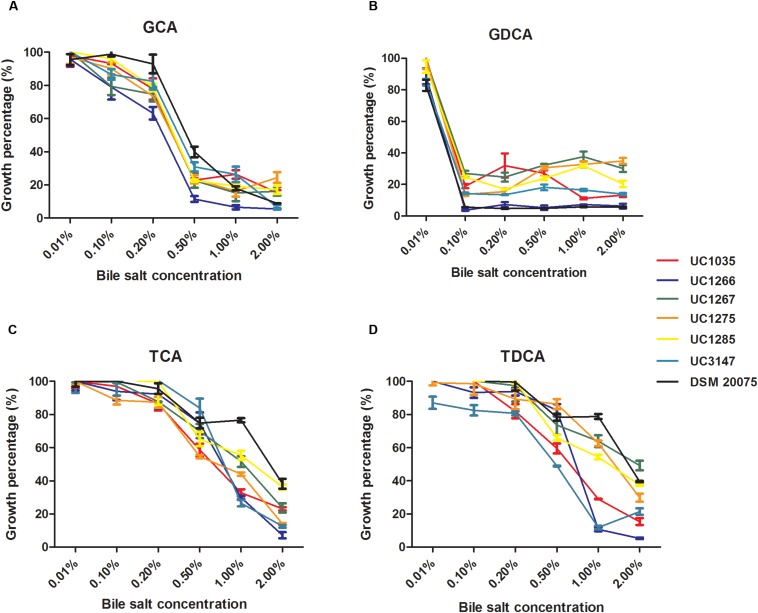
Percentage of growth of the *L. helveticus* strains isolated in this study in the presence of bile salts **(A)** GCA: glycocholic acid. **(B)** GDCA: glycodeoxycholic acid. **(C)** TCA: taurocholic acid. **(D)** TDCA: taurodeoxycholic acid.

Bile salt tolerance was phenotypically evaluated in the six newly sequenced strains. The selected *L. helveticus* strains exhibited similar bile salt tolerance patterns (Figure 6). The strains under investigation were tolerant to all of the tested compounds (i.e., were able to grow at 0.2% of each bile salt), except for GDCA, which inhibited all of the strains at the minimum concentration (0.1%). The effectiveness of GDCA was confirmed in *L. acidophilus* NCFM, which was unable to grow at a concentration higher than 0.05% (McAuliffe et al., 2005). *L. helveticus* UC1267, UC1285, and DSM 20075 demonstrated the highest tolerance, with a survival rate higher than 50% in the presence of TCA at 1% concentration and after exposure to TDCA. Statistical analyses confirmed a significant difference in the bile salt tolerance exhibited by these strains (*P* < 0.05) compared to the others.

**FIGURE 7:**
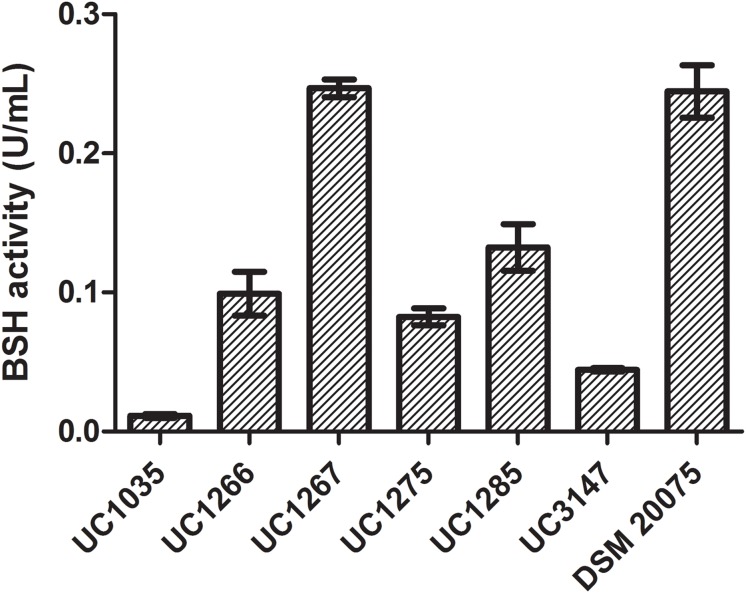
Bile salt hydrolase activity in the *L. helveticus* strains isolated and newly sequenced in this study compared to the reference strain DSM 20075.

The BSH activity in cell extracts of the six *L. helveticus* strains is reported in Figure 7. DSM 20075 and UC1267 had the highest bile salt deconjugation activity and UC1035 had the lowest. A significant difference (*P* < 0.05) was found in the BSH activity of UC1267 and DSM 20075 compared to the other strains. However, in the six strains, the level of BSH was lower than the values reported in literature for other *Lactobacillus* species (Liong and Shah, 2005) and *Bifidobacterium longum* (Tanaka et al., 2000). These findings suggest weak BSH activity and contrast with the results reported by Jiang et al. (2010). In the latter study, BSH activity associated with deconjugation of GDC in *L. helveticus* Lh1 was not detected. Nevertheless, our results are in accordance with Tanaka et al. (1999), who reported a low incidence of BSH activity in typical dairy bacteria species, such as *L. helveticus* and *Lactobacillus delbrueckii.* This outcome is in contrast with isolates from mammalian intestines, which are all BSH-active strains (e.g., *L. acidophilus*, *Lactobacillus gasseri*, and *Lactobacillus johnsonii*). As the standard annotation approach was not effective in identifying the presence of BSH genes and the closely related PVA genes, a dedicated analysis was performed. A hidden Markov model-based procedure (O’Flaherty et al., 2018) targeting the identification of the BSH and PVA gene repertoire was applied. BSH was present in 31 strains (*E*-value < E-99), including all of the newly sequenced strains and DSM 20075, which had high bile salt deconjugation activity. Moreover, the BSH proteins detected in our strains were identical to DSM 20075 in terms of both length and amino acid residues. In contrast, PVA was identified in all strains only with an *E*-value higher than E-99, with the most significant results obtained for 24 strains (including the newly sequenced). Considering other features related to bile salt tolerance, genome annotations revealed the presence of cyclopropane fatty acid synthase encoding gene in all strains. This protein has been related to the bacterial ability of countering high bile acid content typical of the gut environment (Grogan and Cronan, 1997). Finally, transporters of the MFS were previously found to be overexpressed in response to bile exposure in *L. acidophilus* (Pfeiler et al., 2007). Genes encoding for this defense mechanism against inhibitory compounds were also found in the newly isolated *L. helveticus* strains (Supplementary Data S4).

A deep evaluation at the genomic level highlighted features involved in the adaptation to stress induced by gut transit. All of the analyzed strains possess genes responsible for acid tolerance, the heat and cold shock response, and oxidative and general stress (Supplementary Data S4). Proteins involved in establishing proton motive force, such as multisubunit F_0_F_1_ ATPase, Na^+^/H^+^ antiporters, and H^+^/K^+^-exchanging ATPases, are present in all 57 strains and may be putatively involved in pH homeostasis (Senan et al., 2015b). This is expected because some of these proteins play a key role in the basal functioning of the cellular machinery. The phenotypic assay performed to test low pH tolerance (i.e., pH 3) revealed the survival of all the considered strains up to 3 h, except for UC1266 and UC1285 (Supplementary Figure S2). In contrast, genome mining indicated that other genes have a more scattered distribution within the *L. helveticus* species. Among the histidine kinase signal transduction systems, a specific gene (WP_003633555.1) was found to be common in all six newly sequenced strains, as well as another 19 dairy isolates. This HAMP domain-containing histidine kinase can be a useful molecular marker for identification of *L. helveticus* strains associated to dairy ecological niches.

#### Host Adaptation

Antagonism and cooperation for space and resources both contribute to relationships between lactobacilli and other gut microorganisms. For instance, the production of proteins belonging to the cell wall hydrolase/autolysin class can often be related to the control mechanisms of microbial populations sharing the same ecological niche (Salazar and Asenjo, 2007). As suggested previously (Slattery et al., 2010), the presence of maltose-degrading enzymes, along with multiple copies of glucosidase genes, can be considered a putative indicator of gut-adapted microorganisms. Therefore, it is of great interest to highlight the presence of multiple copies (seven to nine copies) of the 6-phospho-β-glucosidase gene in all our strains (Supplementary Data S4), with UC1035 including an extra oligo-1,6-glucosidase gene. These genes were also found in the human-origin probiotic strains D75 and D76 isolated from human gut of a healthy Russian child (Roshchina et al., 2018) and MTCC 5463 isolated from vaginal swab of a healthy Indian female (Senan et al., 2015a). Considering maltose, a gene coding for maltose o-acetyltransferase was found in all of our isolates except for UC1266 (Supplementary Data S4). However, among the newly sequenced strains, only UC1035 encodes a complete maltose transporter (MalEFG), which is also present in the probiotic strain R0052 and in the proposed probiotic strains M92 and MTCC 5463 (Supplementary Data S4).

#### Antibiotic Resistance and Defense Mechanisms

The results of the antibiotic resistance assessment and EFSA breakpoints are presented in Table 1. All strains were highly sensitive to most of the tested antibiotics, demonstrating that they can be used as safe starter strains. The absence of ARG within the chromosomal sequences of our strains and the lack of plasmids were further confirmed by RGI analysis. Thus, the tested strains can be considered safe in terms of antibiotic resistance transferability.

**TABLE 1:** MIC (μg/ml) of antibiotics exhibited by the *L. helveticus* strains isolated in this study.

**Strain**	**Gm**	**Km**	**Sm**	**Nm**	**Tc**	**Em**	**Cl**	**Cm**
UC1035	≤0.5	16	2	2	2	0.06	≤0.03	2
UC1266	≤0.5	16	1	2	2	0.03	≤0.03	2
UC1267	≤0.5	≤2	≤0.5	1	0.5	≤0.016	≤0.03	0.5
UC1275	≤0.5	4	≤0.5	≤0.5	1	0.06	0.06	2
UC1285	≤0.5	4	1	1	1	0.03	≤0.03	1
UC3147	≤0.5	≤2	2	2	1	0.03	0.06	1
DSM 20075	≤0.5	≤2	≤0.5	≤0.5	2	0.12	0.06	4
EFSA cutoff values	16	16	16	–	4	1	1	4

*Antibiotics abbreviations: Gm, gentamycin; Km, kanamycin; Sm, streptomycin; Nm, neomycin; Tc, tetracycline; Em, erythromycin; Cl, clindamycin; Cm, chloramphenicol.*

Regarding the CRISPR-Cas system, RAST annotation identified CRISPR-associated genes (Cas) in four of the newly sequenced strains, with UC1035 having five genes and UC1275, UC1285, and UC3147 having two genes. The highest number of genes in this category was exhibited by the H9 strain (seven genes), whereas ATCC 10386, CGMCC 1.1877, CIRM-BIA 101, DSM 20075, LH99, and M3 all contained six genes. Additionally, CRISPRCasFinder identified CRISPRs in all newly sequenced strains, with UC1266 showing the highest number (three). Strains UC1275, UC1285, and UC3147 presented two CRISPRs, whereas UC1035 and UC1267 had only one repeat (Supplementary Data S3). Among the other 51 strains analyzed, the highest CRISPRs content was exhibited by D75 and D76 (four sequences), followed by H9 and KLDS1.8701 (three sequences); all these strains have proven probiotic capabilities (Chen et al., 2015; Li et al., 2017; Roshchina et al., 2018).

The presence of bacteriocin-producing genes was also evaluated, because it is an important probiotic trait for bacterial competition in a complex microbial environment, such as the human gut. Bacteriocins may directly inhibit the invasion of competing strains or pathogens, or modulate the composition of the microbiota and influence the host immune system by enhancing human health (Dobson et al., 2012). The genomic analysis highlighted the presence of genes encoding bacteriocins. Specifically, enterolysin A was found in four of our strains (UC1035, UC1266, UC1285, and UC3147), as was helveticin (UC1266, UC1267, UC1275, and UC3147; Supplementary Data S4), both belonging to class 3 bacteriocins, which are high-molecular-weight and heat-labile antimicrobial proteins (Alvarez-Sieiro et al., 2016). Additional bacteriocin genes were found in our strains, with UC1266, UC1267, UC3147, and UC1285 having the highest content (four and three genes, respectively; Supplementary Data S2, S4). The scattered distribution of bacteriocin-encoding genes in the examined strains was also confirmed by the association of these genes with strain-specific regions, as shown by the pangenome analysis. Regarding the bacterial secretion system, bacteriocin ABC transporters were found only in UC1266 (two genes) and UC1285 (three genes) (Supplementary Data S2). Nevertheless, some of the genes encoding components of the Sec-dependent transporter system were found in all of the strains except for SecG, which was absent in UC1275 (Supplementary Data S2). The Sec-dependent transporter system has also been found to be involved in bacteriocin secretion (Herranz and Driessen, 2005).

## Conclusion

This study describes a genome-centric strategy to select strains of *L. helveticus* for probiotic purposes. To address this intent, a large-scale genomic analysis was performed by considering all of the publicly available genomic sequences, along with six newly sequenced strains isolated from natural whey cultures. The genome-based investigation of probiotic features was also supported by specific phenotypic assays.

Pangenome analysis revealed gene clusters specifically present in the new isolates, including enzymes responsible for folate biosynthesis in UC1266 and UC1267. The correlation between BSH activity and bile salt resistance was confirmed regarding TCA and TDCA. Indeed, the strains with the highest enzyme activity (DSM 20075 and UC1267) were also the most resistant to these bile salts. However, considering GCA and GDCA bile salts, this correlation fails, indicating the involvement of other mechanisms of resistance to bile salts in addition to BSH activity. This hypothesis is also supported by the encoded BSH proteins in the new isolates with low bile salt tolerance. Indeed, their BSH proteins are identical in both length and amino acid residues to those of the tolerant strains DSM 20075 and UC1267. Concerning the safety profile of the new strains, the lack of antibiotic resistance, both phenotypically and genotypically, was assessed. Two strains, UC3147 and UC1285, were characterized by the highest number of bacteriocin genes among the new isolates. Considering EPS production, the ropy phenotype was detected exclusively in UC1275, probably related to the dTDP-rhamnose reductive pathway. Finally, the presence of maltose-degrading enzymes and multiple copies of the 6-phospho-β-glucosidase gene in our natural whey culture isolates indicates the capability to metabolize sugars other than lactose.

Considering both the phenotypic and genotypic properties of the investigated *L. helveticus* strains, more pronounced adaptability to the gut environment was shown in the newly sequenced strain UC1267 and in DSM 20075. Specifically, the highest BSH activity and bile salt tolerance, the presence of maltose-degrading enzymes, and multiple copies of glucosidase genes highlighted their potential to survive in the GIT. Moreover, the presence of multiple genes encoding bacteriocins and a complete pathway for folate production could also be involved in the health-promoting effects on the host. Further studies will be necessary to test their probiotic efficacy *in vivo*.

## Author Contributions

All authors performed the analysis, prepared the manuscript, and contributed to editing and critical reviewing.

## Conflict of Interest Statement

The authors declare that the research was conducted in the absence of any commercial or financial relationships that could be construed as a potential conflict of interest.

## Footnotes

1 https://www.ncbi.nlm.nih.gov/genome/microbes/

2 https://sourceforge.net/projects/lactobacillus-helveticus/

## Supplementary Material

FIGURE S1 Genomic alignments. The genomes of the six *L. helveticus* strains sequenced in the present study were aligned considering CAUH18 as a reference. Some relevant genomic regions identified in the six strains and absent in CAUH18 are highlighted and a summary of the genes included is reported in the right part of the figure. Local co-linear blocks (syntenic regions) identified are represented by different colors.

FIGURE S2 Low pH tolerance (pH 3) of the six newly sequenced *L. helveticus* strains and DSM 20075.

DATA S1 Dataset A. “Newly sequenced strains” worksheet. Genome properties and details regarding the six newly sequenced *L. helveticus* strains. Dataset B. “NCBI strains” worksheet. Genome properties and details regarding the 51 *L. helveticus* strains publicly available.

DATA S2 Dataset A. “Genes distribution” worksheet. Gene “presence/absence” in all 57 *L. helveticus* strains determined using Roary and correspondent coverage calculation based on raw sequences alignment. Dataset B. “SEED annotation” worksheet. Number of genes identified for each SEED category in all *L. helveticus* strains.

DATA S3 Dataset A. “BSH hmm results” worksheet. Results of the HMM analysis performed to identify genes encoding bile salt hydrolase (BSH). Dataset B. “PVA hmm results” worksheet. Results of the HMM analysis performed to identify genes encoding Penicillin-V Acylase (PVA). Dataset C. Results of the analysis performed using CRISPRCasFinder to identify CRISPR systems.

DATA S4 Dataset A. “Adhesion and aggregation genes” worksheet. Results from genome mining as presence/absence of genes known to be related to adhesion and aggregation features in all *L. helveticus* strains. Dataset B. “Host adaptation” worksheet. Results from genome mining as presence/absence of genes known to be related to host adaptation features in all *L. helveticus* strains. Dataset C. “Mobile genetic elements” worksheet. Results from genome mining as presence/absence of genes known to be related to mobile genetic elements features in all *L. helveticus* strains. Dataset D. “Stress response” worksheet. Results from genome mining as presence/absence of genes known to be related to stress response features in all *L. helveticus* strains. Dataset E. “Highlight stress response” worksheet. Genes responsible of stress response are grouped in general categories. Dataset F. Results of Bagel4 for the UC strains.
