# Supplementary material for: Genomic Comparison of Lactobacillus helveticus Strains Highlights Probiotic Potential
Source: Front Microbiol. 2019 Jun 26;10:1380. doi: 10.3389/fmicb.2019.01380 (PMC6606699; doi:10.3389/fmicb.2019.01380)
Supplement: FIGURE S1 — Genomic alignments. The genomes of the six L. helveticus strains sequenced in the present study were aligned considering CAUH18 as a reference. Some relevant genomic regions identified in the six strains and absent in CAUH18 are highlighted and a summary of the genes included is reported in the right part of the figure. Local co-linear blocks (syntenic regions) identified are represented by different colors. [file Data_Sheet_1.PDF]

Genomic Comparison of *Lactobacillus helveticus* Strains Highlights Probiotic Potential

Alessandra Fontana, Irene Falasconi, Paola Molinari, Laura Treu, Arianna Basile, Alessandro Vezzi, Stefano Campanaro and Lorenzo Morelli

Supplementary figures

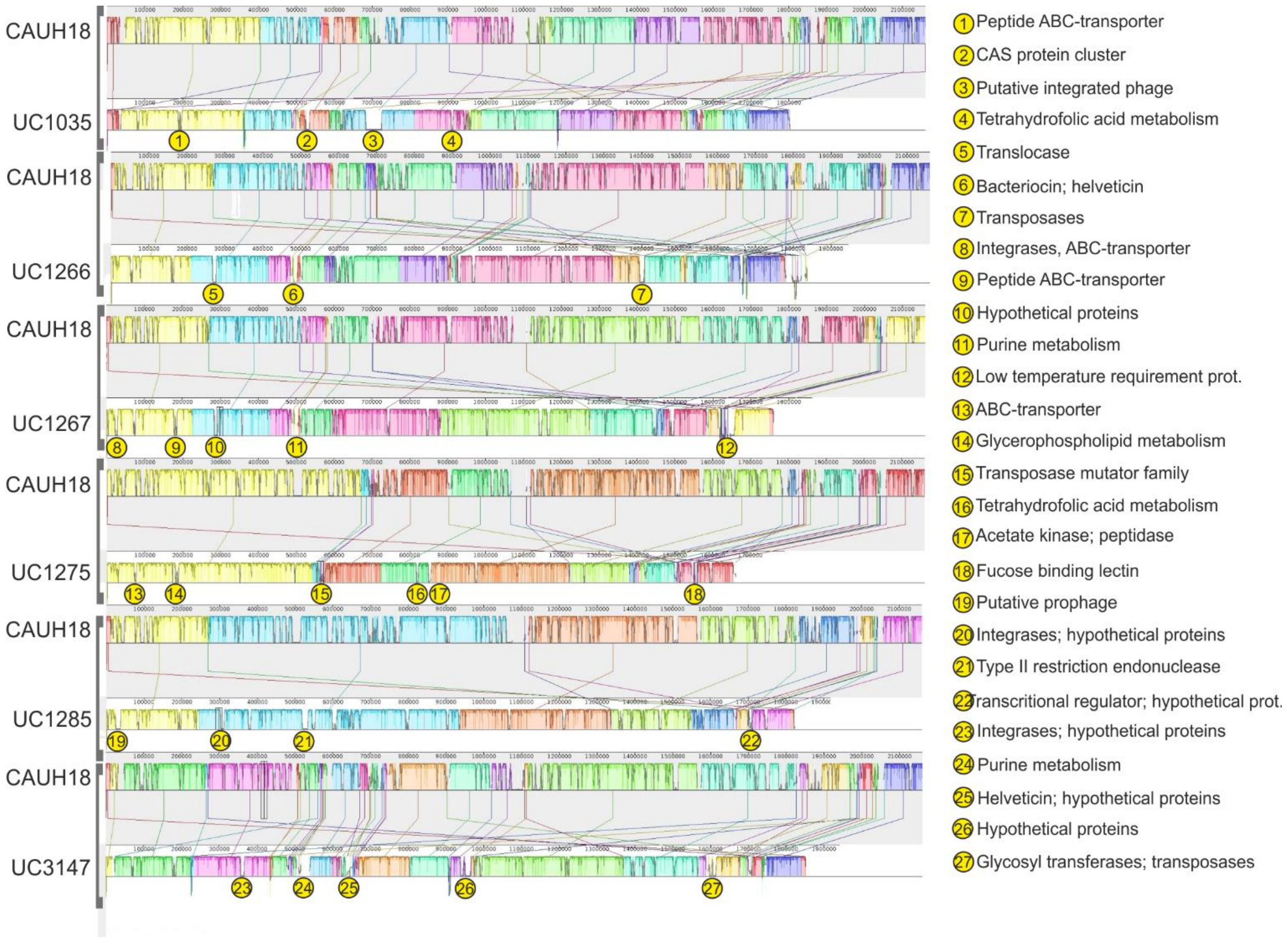

Supplementary figure S1. Genomic alignments. The genomes of the six *L. helveticus* strains sequenced in the present study were aligned considering CAUH18 as a reference. Some relevant genomic regions identified in the six strains and absent in CAUH18 are highlighted and a summary of the genes included is reported in the right part of the figure. Local co-linear blocks (syntenic Regions) identified are represented by different colors.

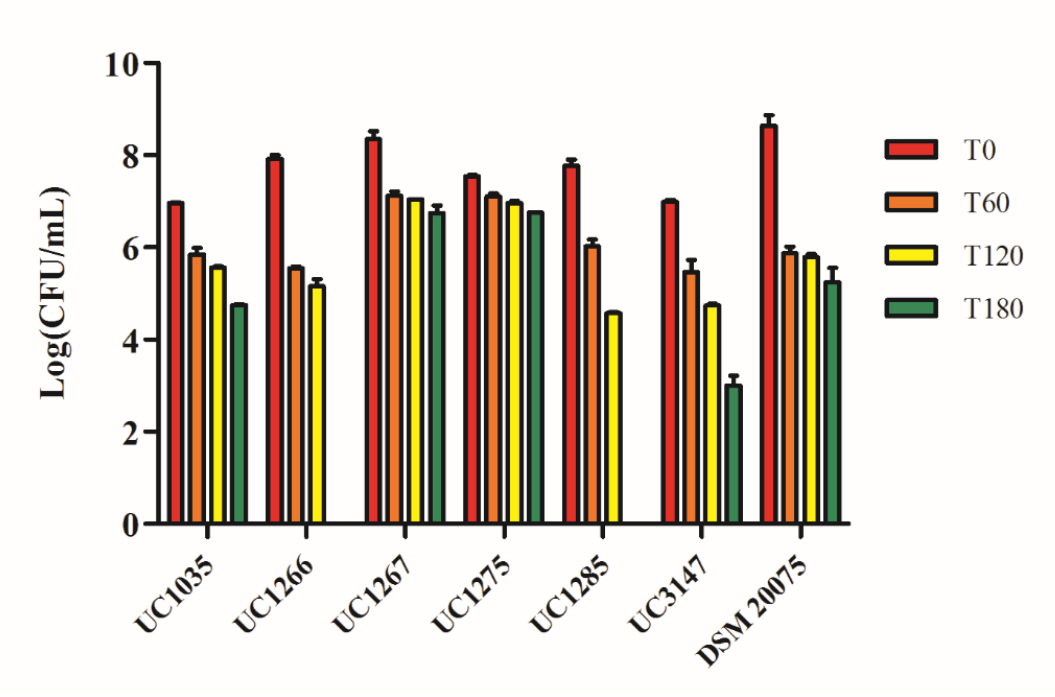

Supplementary figure S2. Low pH tolerance (pH 3) of the six newly sequenced *L. helveticus* strains and DSM 20075.
